# Evaluating the transport, health and economic impacts of new urban cycling infrastructure in Sydney, Australia – protocol paper

**DOI:** 10.1186/1471-2458-13-963

**Published:** 2013-10-17

**Authors:** Chris Rissel, Stephen Greaves, Li Ming Wen, Anthony Capon, Melanie Crane, Chris Standen

**Affiliations:** 1 Sydney School of Public Health, University of Sydney, 92-94 Parramatta Road, Camperdown, Sydney, NSW 2050, Australia; 2 Institute of Transport & Logistics Studies (C13), University of Sydney Business School, Sydney, NSW 2006, Australia; 3 Sydney and South Western Sydney Local Health Districts, Level 9, King George V Building, Sydney, NSW 2050, Australia; 4 University of Canberra, University Drive, Bruce Australian Capital Territory, Canberra 2617, Australia

**Keywords:** Bicycle, Infrastructure, Physical activity, Cycling, Urban design

## Abstract

**Background:**

There are repeated calls to build better cycling paths in Australian cities if the proportion of people cycling is to increase. Yet the full range of transport, health, environmental and economic impacts of new cycling infrastructure and the extent to which observed changes are sustained is not well understood. The City of Sydney is currently building a new bicycle network, which includes a new bicycle path separated from road traffic in the south Sydney area. This protocol paper describes a comprehensive method to evaluate this new cycling infrastructure.

**Method:**

A cohort of residents within two kilometres of the new bicycle path will be surveyed at baseline before a new section of bicycle path is built, and again 12 and 24 months later to assess changes in travel behaviour, sense of community, quality of life and health behaviours. Residents in a comparable area of Sydney that will not get a new separated bike path will act as a comparison group. At baseline a sub-set of residents who volunteer will also take a small GPS device with them for one week to assess travel behaviour.

**Discussion:**

This research should contribute to the advancement in evaluation and appraisal methods for cycling projects.

## Background

There are clear personal, social and environmental benefits of cycling, and these benefits increase as more people cycle [1]. However, while the number of people cycling in Australia has been increasing in the last few years, they still only constitute around one per cent of all daily trips [2]. While roughly comparable to North America and the UK, this is well below cycling levels in Northern continental Europe, where land use and transport policies have been more supportive of cycling and discouraging of car use [3]. Recently, however, there have been signs of a shift in thinking about cycling, reflected in the setting of targets at all three tiers of Australian government supported (in principle at least) by infrastructure, education, and other programs supporting cycling. For example, the Australian National Cycling Strategy has set a goal to double the number of people cycling by 2016 [4]. The government of New South Wales, in its State Plan, has set a target to increase the mode share of bicycle trips made in the Greater Sydney region, at a local and district level, to 5% by 2016 [5], and published a Bike Plan which outlines how this target can be achieved [6]. Individual local government areas have also set targets, such as the City of Sydney’s Cycle Strategy and Action Plan 2007–2017, which has a target of 10% of trips to be made by bike by 2016 [7].

The motivation to encourage wider uptake of cycling has come from multiple directions reflecting the multiple benefits of cycling. Transport and urban planning representatives have pursued the transport benefits (e.g., reduced congestion, increased non-motorised transport); environmental groups have focused on the environmental benefits (e.g., reduced greenhouse gases, improved air quality, less noise); while public health and social groups have focused on the health benefits (tackling physical inactivity and associated consequences), and wider community benefits (e.g., liveability, sense of community). There is also increasing calls for interventions and strategies to address both environmental and public health issues through transport strategies that promote active transport [8].

Despite this, current cycling infrastructure evaluation tools in Australia do not yet support such a multi-faceted approach for (at least) three primary reasons. Firstly, while Australia is arguably a world-leader in the evaluation of travel-behaviour change programs (such as TravelSmart), evaluation criteria are largely restricted to assessments of changes in vehicle kilometres of travel (VKT) and greenhouse gas emissions [9]. Where evaluations have considered other dimensions, such as health, these have been done using different methods/criteria with limited efforts to bring them together with the transport or economic dimensions. Secondly, there is a distinct lack of convincing, quantifiable evidence on the ‘before’ and ‘after’ impacts per se of cycling infrastructure interventions world-wide [10]. The net result is that we have an incomplete picture of what might be best termed the ‘full societal impacts’ of cycling infrastructure policies. Thirdly, the before and after studies that have been done have rarely assessed whether observed changes in behaviour are sustained over the longer term (up to 24 months) and there has to our knowledge been no longer term follow-up of the health outcomes from changes to cycling environments.

The evaluation of cycling infrastructure has been considered from the transport/environmental, health and economic perspectives. Each of these is examined below.

### Transport/environment

Evaluations of transport infrastructure largely focus around the impacts on travel times, vehicle kilometres travelled (VKT), and mode choice. Environmental measures have become a crucial component of transport evaluations, particularly through the impacts on air quality, greenhouse gases, and noise. Measures of travel behaviour using paper diaries or surveys are limited by recall problems and are generally only feasible for a very short period of time. This can be a significant weakness, as travel can be highly variable from day to day, particularly between weekends and weekdays [11]. Technologies such as global positioning systems (GPS), smartphones and online travel diaries permit much more accurate measures of travel behaviour allowing (crucially) small changes in behaviour to be detected [12]. The latest personal GPS devices are low-cost, capable of storing weeks of travel data, and have taken on the characteristics of mobile phones, enabling them to be carried unobtrusively by large numbers of participants. Coupled with this, through integration with web-based technology, it is now feasible to provide information/feedback to participants on their travel and prompt them for additional data, such as the purpose of travel.

Smartphones in theory have a number of advantages over personal GPS devices. As well as having integrated GPS receivers, they can track travel behaviour using their in-built accelerometers, mobile network positioning and Wi-Fi positioning. In addition, data can be uploaded in real-time or in frequent batches, unlike personal GPSs which must be connected to a computer. Unfortunately current battery technology is not adequate for smartphones to have their GPS receivers switched on all day. Online travel diaries have a number of advantages over paper-based ones. In particular, participants can be prompted for additional information about their trips. For example, where they say they went somewhere by train, they can be reminded to provide information about their access and egress trips.

### Health

Little attention has been paid to health or physical activity variables and no research (to our knowledge) has examined the effect of transport on quality of life indices. Some new assessment tools (e.g., HEAT) [13] have included measures of mortality but this is a very crude measure of health, and not viable with small area analyses.

Quality of life is an important health measure, increasingly used to assess the impact of health intervention programs. Clearly transport initiatives can affect quality of life, but changes to quality of life as a result of new transport infrastructure have never been systematically quantified and documented. Research into the effect of urban design and development on physical activity and other health indicators has increased dramatically in the past decade, but clear causal links between positive changes in the cycling environment and health have been elusive. In 2003, a new Sydney RTA built cycle and walk-way, the Parramatta-Liverpool Rail-Trail was evaluated, one of the first such studies internationally [14]. With only minimal community promotion of the Rail-Trail, only moderate increases in use were found.

In the same region of south western Sydney, a demonstration grant to promote use of existing bicycle paths and explore associations with increased physical activity, found a significant increase in use of the cycling infrastructure, but did not detect increases in physical activity [15]. This may have been because the cycling infrastructure was already in place, and was primarily used for recreation. Early results from the demonstration cycling towns as part of the Cycling England project have reported increases in cycling and increases in population levels of physical activity [16]. A US study of cycling found that sixty per cent of the cyclists surveyed rode for more than 150 minutes per week during the study and nearly all of the cycling was for utilitarian purposes, not exercise [17]. Other research from the US has found positive associations between miles of bicycle pathways per 100,000 residents and the percentage of commuters using bicycles [18] and that new bicycle lanes in large cities will be used by commuters [19]. A significant barrier to encouraging more people to cycle, particularly beginner or non-confident riders, is concerns about interacting with motor vehicles and the desire for separated bicycle paths [20]. Separate bicycle paths may also be important for encouraging women to travel by bicycle [21]. In areas where cycling culture is not established (such as Sydney) the evidence is unclear and there is still uncertainty whether changes to the built environment and other transport interventions improve cycling participation [10].

### Economics

Investment in ‘cycling specific’ infrastructure has consistently had positive results, generally because the value of health benefits can be substantial and dwarfs the initial construction costs. For example, the NSW Roads and Traffic Authority reported a ratio of 1.3:1 when calculating the cost-benefit ratio of building missing links in its cycle network, using conservative assumptions [22], and a specifically focused analysis of the Inner Sydney Regional Bicycle Network found the cost-benefit ratio was 3.88:1 [23].

Traditional cost-benefit analyses of cycling infrastructure do not generally consider the wider/indirect economic impacts, including impacts on local business and retail establishments. The very limited evidence that does exist is generally favourable for cycling infrastructure. For instance, an inner city Melbourne study found that while car users averaged more overall spending per hour than bike riders, the small area of public space required for bike parking means that each square metre allocated to bike parking generated $31 per hour, compared to $6 generated for each square metre used for a car parking space [24]. Anecdotal reports from the City of Sydney suggest that new businesses have started along new separated bike paths, adding to the local economy and reducing VKT to other retail centres. Additional pedestrian traffic resulting from new cycling infrastructure has also been reported, with further health benefits potentially accruing.

### Research aims

Within Australia, the City of Sydney has an ambitious cycling plan, calling for ten per cent of trips to be made by bicycle by 2016. The main component of achieving this target is a comprehensive bicycle network separated from motor vehicle traffic, which is being constructed over a number of years [7]. To support this infrastructure, a carefully planned behavioural strategy will also be implemented across the entire City [25]. In early 2014 a critical section of this network will be built, which is anticipated to lead to significant increases in cycling. However, impacts of the new path will be assessed using traditional evaluation methods such that the full societal impacts will be known only partially.

With this in mind, the aims of the proposed research are:

1. To assess changes in i) transport and environmental outcomes (e.g., cycling behaviours and frequency among local residents, bicycle kilometres travelled [BKT], greenhouse gas emissions estimates), ii) health indicators (e.g., physical activity, quality of life [WHOQoL], sense of community and community cohesion) among local residents associated with use of the cycling infrastructure, participation in cycling promotion activities), and iii) wider economic benefits associated with new cycling infrastructure, including changes in local retail dynamics and retail related travel.

2. To strengthen traditional cost-benefit analyses of cycling infrastructure by developing an easy to use multi-criteria evaluation tool using readily collectable data, which considers the transport, environment, health and wider economic impacts of proposed cycling infrastructure investments.

## Method/Design

The proposed approach involves 1) assessments of the local community before and after (12 and 24 months) the construction of a separated bicycle path to assess changes in travel, environmental, health and wider economic outcomes, compared with a similar community with no new cycle path; and 2) development of a new appraisal tool for evaluating cycling projects, which utilises this richer empirical evidence on actual (as opposed to hypothetical) outcomes. The research has been approved by the Human Research Ethics Committee, The University of Sydney (protocol number 2012/2411). Participation in the on-line survey is considered consent to participate, and separate written informed consent for participation in the study will not be obtained from participants. All participants are adults aged 18 years or over.

### Phase 1: quasi-experimental design

**Figure 1:**
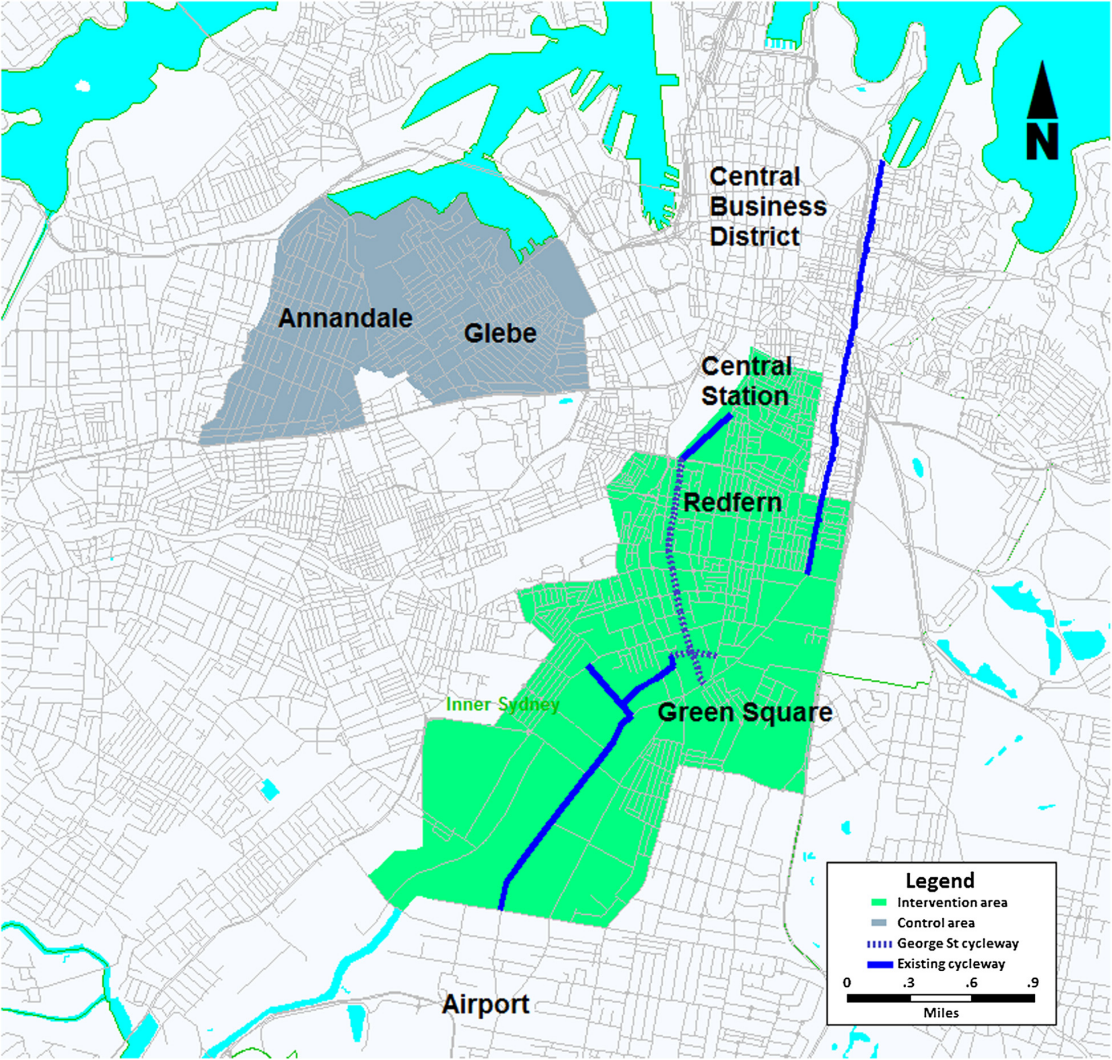
The interventiona and comparison area.

The before-and-after evaluation will be conducted as a quasi-experimental study including regular bicycle observational counts, plus a cohort study with three data collection points in two areas: an intervention area with a new separated bicycle path and a comparison area. The intervention area is defined as the cycling catchment area (less than two kilometres) for the planned new bicycle path in south Sydney (see Figure 1). This design has been used previously by Rissel and Wen [15]. There are approximately 18,000 residents in the intervention area (allowing for growth since the 2006 census), with 30% born overseas, and two-thirds (65%) aged between 18 and 55 years, and thus within the target population parameters. The comparison area will include a demographically similar part of Sydney where no bicycle paths are planned during the same period, in terms of population size and density, occupation, income, ethnicity, proximity to CBD and current cycling infrastructure. It is possible that the residents in the comparison area may cycle on the new City of Sydney bicycle paths. While this exposure is impossible to control in a community context, baseline assessments of cycling and walking can take into account any differences between the intervention and control areas regarding physical activity patterns. Analyses will test for the effect of exposure to the intervention.

#### Study population

The target population will be residents of the intervention or comparison area. Eligible respondents will be aged 18–55 years, speak sufficient English to participate, have ridden a bicycle in their lifetime, have no disability that prevents them from riding a bicycle, and are not planning on moving away from their neighbourhood. Study participants will be recruited using an online sampling methodology, an approach which has been successfully used in other cycling cohort studies [26]. Members of an existing online panel managed by a contracted market research company will be invited to participate directly via email. Should additional respondents be required in our defined geographical area, further eligible participants will be identified via a computer assisted telephone interview (CATI) panel supplemented by Random Digit Dialing (RDD), followed by an email invitation if they are willing to participate which will direct them to an online survey. In the case of the CATI panel sample, multiple in-scope respondents will be invited to participate where they are present in each household.

#### Data collection

Primary data will be collected using an on-line questionnaire plus an online travel diary to be completed over seven days. In addition, approximately half the sample will be offered the opportunity to take a small personal GPS device to record detailed travel/activity behaviour and potentially infer energy expenditure for establishing changes in levels of physical activity. This is the most cost-effective approach to data collection, particularly given the large number of apartment complexes in the inner-city, which severely restricts direct access to householders through door-knocking. Piloting of the on-line instruments, with incentives for completion, led to a high completion rate.

The baseline questionnaire (approximately 20 minutes) will be conducted in August/September (the southern hemisphere spring) 2013 before the bicycle path is built, and respondents will be re-contacted 12 and 24 months later. The aim is to complete the data collection before the spring school holidays in late September but in the event recruitment problems are encountered it may be necessary to conduct a further wave of data collection after the school holidays to reach the required numbers. The content of the baseline and the follow-up questionnaires will be similar. Socio-demographic characteristics (including age, gender, educational attainment, income, marital status, presence of children in the household, bicycle and car ownership) will be asked at baseline. In the follow-up questionnaires, any changes in these attributes will be ascertained with additional questions regarding respondents’ experiences of the newly built bicycle paths in their neighbourhood. The physical activity questions are based on the validated Active Australia questionnaire [27].

The online travel diary that will be used in this study incorporates many innovations designed to make it simple, relatively quick and engaging for participants to complete, while capturing the key travel information required for the study (i.e., trip origins, destinations, start and end times, modes used, purposes). A particular concern for this study is the collection of access/egress travel to/from public transport modes, which tends to be forgotten. This has been addressed through a leg-based approach in which participants are ‘forced’ to recall details of the access/egress mode including duration. Pilot testing of the diary, showed a 90% completion rate [defined as completing all seven days] of the diary with on average each trip taking around 90 seconds to complete with exit surveys of participants suggesting a very positive response to the diary. An additional innovation planned for the main data collection phase is an optional capability for participants to view an electronic map of their travel collected via a Smartphone app developed by the project team while completing the diary, the purpose of which is to help with recall. The personal GPS devices have previously been used successfully in the monitoring of personal travel in several large-scale projects [17] including the monitoring of cycling [28]. They are small and portable (similar to a mobile telephone), capable of storing up to a month’s worth of travel data, and go for around three days between charges subject to usage. Participants will be given the device for a one week period concurrent with the diary period, the primary purpose of which is to develop correction factors for the self-reported information from the diary [9].

Based on our previous experiences in longitudinal behavioural change experiments [29] it is probable there will be changes in the personal circumstances of some participants that may impact their behaviour (e.g., changing job, moving house, having a baby etc.). To the extent that is possible, we will control for these factors by asking people at each time-point, what has changed since the last survey wave and incorporating this information in the analysis of the quantitative measures of health and travel.

#### Sample size

Sample size calculations are based on two sets of comparisons. The first is the detection of changes in bicycle kilometres of travel (BKT). Using general BKT figures, and based on a travel target of a 12% increase in bicycle BKT, approximately 343 people are required in each area with type I error = 0.05 and Power = 0.8. The second outcome is increases in time spent cycling in the previous week. From a previous study, the mean time spent cycling was 41 min (SD = 108) per week. To detect a difference of 30 minutes per week between intervention and control groups, 187 cyclists in each group would be needed. As this is lower than the sample size required for the detection of changes in the prevalence of sufficiently active cyclists (above) the larger sample will be needed. Further adjustment for an assumed 15% attrition rate at both the 12 and 24 month follow-up surveys (based on experience with another cohort [9] would require a baseline sample of around 480 respondents in each area.

#### Key outcome measures

##### Health measures

Physical activity (PA) behaviour: Total time cycling and walking per week (based on the national Active Australia questionnaire and the on-line travel diary); total time physically active, per cent rated as sufficiently active (sufficient to confer health benefit if total time is greater or at least 150 minutes).

Awareness and usage of bicycle paths: Unprompted and prompted awareness of bicycle paths, and usage based on number of times respondent used any City of Sydney bicycle path or (post construction) the new cycleway during the past week and month. Awareness of cycling promotion activities will also be recorded.

Potential intervening causal or mediating measures will be examined: including perceived safety of bicycle path; perceived distance from bicycle path (to the nearest 100 m); perceived barrier index (access, time, routes); and perceived neighbourhood characteristics.

Active commuter: if respondent commutes to work, part way or the whole way by walking or cycling.

Sense of community: The Sense of Community Index (SCI-2) [30] will be used to measure sense of community, and community cohesion using a comprehensive framework for transport planning developed by Litman that includes valuing community cohesion and social connectedness [31].

Quality of Life: Developed by the World Health Organisation, the WHOQOL-BREF was developed as a 26-item international cross-culturally comparable quality of life assessment instrument [32]. The Australian version of the WHOQOL-BREF will be used as question item scales have been adapted for an Australian audience, while retaining its wider generalisability.

##### Transport measures

The online travel diary and GPS-based information will be used to infer key travel information over a seven day period. These include trip rates, trip times, mode shares, kilometers of travel/mode, trip purposes, and proportion of bicycle travel using cycling paths, which can be compared over the three time-periods (baseline, after 12 month, after 24 months) for evidence of a change. In addition, changes in vehicle kilometers of travel (VKT) will be used to estimate changes in environmental metrics, primarily greenhouse gas emissions (11).

##### Cycle path use

The City of Sydney will conduct regular six monthly counts of cyclist traffic at key intersections during the entire study period. This is a well developed observational method used consistently by the City of Sydney and used for national bicycle counts conducted by Bicycle Victoria. In addition, in follow-up data collection waves we will conduct more detailed analyses of how cyclists in the sample actually use the cycle-paths based on the GPS data together with appropriate questions on their experiences.

##### Economic measures

Actual costs associated with the building of the new bicycle path will be obtained from the City of Sydney. Actual benefits will be calculated based on traditional cost-benefit analysis (CBA criteria resulting from any changes in travel mode, including standard health benefits, reduced noise costs, reduced air pollution, reduced greenhouse gases, reduced congestion, reduced vehicle operating costs, and car parking costs savings. Sensitivity analysis will be conducted with the higher quality GPS travel and physical activity measures to calculate changes in the benefit values. Qualitative criteria will also be assessed, including amenity values, improved access, community engagement and interaction and increased liveability [22]. Based on these data, a new index or set of measures of the value of cycling based on travel mode and physical activity (adjusting for other costs and benefits) will be developed.

Changes in the local economy will be examined with a qualitative sub-study to examine the impact of the bicycle paths on residents and local retailers that may be affected by the building of a new bicycle path. Qualitative interviews will be conducted at baseline and again at 24 months. The methodology will include questions about travel and expenditure used in a Melbourne study of visitors to a retail area and asked about travel mode, expenditure and reason for their trip [24].

#### Statistical analysis

Analyses of questionnaire data will test the significance of pre-post-follow-up changes in the cohort and include paired t-tests for continuous variables and McNemar’s test for categorical measures. Comparisons between the groups will also be made by Pearson chi-square tests or t-tests. General linear regression will be used for continuous outcome measures and logistic regression will be used to analyse the categorical outcome measures to determine the intervention effect. The analyses will assess the differences in the outcome measures from baseline to post survey by level of intervention, by distance of home from the bicycle path and other potential explanatory measures adjusting for socio-demographic characteristics and baseline level of activity. To detect whether there is a significant increase in cycling activity in the monitored area the bicycle counts for before and after the campaign will be calculated for each location, and then stratified by weekends, season and by periods (pre-launch, post-launch, intervention period and post intervention).

### Phase 2: development of multi-criteria appraisal framework

The data from Phase 1 will be used to develop a simple-to-use appraisal tool for cycling interventions that incorporates health, travel, environmental and wider community/economic outcomes. The starting point and our bench-mark for comparison will be existing appraisal tools used in New South Wales [22,23] and overseas [33]. Briefly, existing appraisal methods estimate the demand for a new facility (typically using borrowed assumptions or in some cases a stated preference survey), apply costs to the intervention (e.g., bicycle path) and assign monetary values equating to the costs and benefits that accrue to travellers on a per kilometre basis as a direct result of that intervention.

Current approaches suffer from many limitations that undermine the appraisal of cycling projects world-wide, particularly around i) the estimation of demand and diversion rates (i.e., those who switch from other modes to cycling), ii) the parameters used (e.g., health benefits, travel time implications) and iii) the failure to consider the indirect benefits to the wider community and economy. The outcomes of Phase 1 will be used to address directly all three of these limitations by using the travel diary and GPS information to validate/update existing logit-based mode-choice model parameters and diversion rates, travel time and energy expenditure parameters. The outcomes of the quality of life survey and retail surveys will add an indirect benefit capability to the framework.

## Discussion

This research will provide a significant advancement in evaluation and appraisal methods for cycling projects. Current methods are essentially an adaptation of motorised transport techniques, which are largely focused on transport time/cost savings with unconvincing efforts to incorporate aggregate-level health impacts [9]. There is considerable capacity to increase both the absolute numbers of cyclists and cycling frequency. For instance, in Sydney, one quarter of car trips are less than two kilometres, while one half of car trips are less than five kilometres, distances that are (in theory at least) amenable to cycling [7]. Only one per cent of Sydney’s population cycles each day, but 30 per cent of Australians have cycled in the past year [34]. Spending on cycling infrastructure constitutes less than one per cent of the total spent on transport infrastructure in Australia, yet it is estimated that it saves the economy around $64 million in reduced traffic congestion [1]. Current regular cycling is estimated to save $227 million annually in reduced health costs [1].

There are several innovative aspects of this research. First, it will represent the first effort in Australia to develop a framework for assessing cycling infrastructure investments, which jointly considers transport, environmental, economic, and health impacts. Second, it represents one of the largest and most comprehensive assessments worldwide to study the pre- and post-impacts of cycling infrastructure on changes in travel, physical activity, and quality of life outcomes of the population in the intervention area. The vast majority of studies assessing new cycling infrastructure are done either by ‘borrowing’ assumptions from other studies or surveying people about whether they will use the new infrastructure using stated preference (SP) techniques [15]. Such evaluations are rarely validated (other than through the weak proxy of bicycle counts), which tell us nothing about whether actual changes (Revealed Preferences – RP) in behaviour match those in the SP setting. Where RP validation of SP results has been done, it is very evident that SP suffers from hypothetical bias in that people do not follow through with what they say they will do [29]. Third, while previous studies have used online travel diaries and GPS for monitoring travel, the linking of travel diary and GPS data to measures of physical activity (energy expenditure) has been poorly done with no reliable algorithms available. This will provide significantly more accurate information for both transport and health outcomes than is currently available. Internationally there is no substantial research linking transport with objective measures of quality of life and changes in the active transport environment. Finally, the long follow-up of health and economic outcomes of changes in transport is internationally significant.

## Competing interests

The authors declare that they have no competing interests.

## Authors’ contributions

CR and SG conceived of the study, CR, SG, LMW and TC participated in its design, the submission of the successful grant application, and all authors drafted the manuscript. All authors read and approved the final manuscript.

## Pre-publication history

The pre-publication history for this paper can be accessed here:

http://www.biomedcentral.com/1471-2458/13/963/prepub
